# Measuring optokinetic after-nystagmus: potential for detecting patients with signs of visual dependence following concussion

**DOI:** 10.1007/s00415-020-10359-8

**Published:** 2020-12-26

**Authors:** Giovanni Bertolini, Fausto Romano, Dominik Straumann, Katharine Keller, Antonella Palla, Nina Feddermann-Demont

**Affiliations:** 1 Swiss Concussion Center, Schulthess Clinic, Zurich, Switzerland; 2 Department of Neurology, University Hospital and University of Zurich, Frauenklinikstrasse 26, CH-8091 Zurich, Switzerland; 3 University of Zurich, Zurich, Switzerland; 4 Clinical Neuroscience Center, Zurich, Switzerland

**Keywords:** Velocity storage, Self-motion perception, Human, Vestibulo-ocular reflex, Psychophysics

## Abstract

Concussed patients with chronic symptoms commonly report dizziness during exposure to environments with complex visual stimuli (e.g. supermarket aisles, busy crossroads). Such visual induced dizziness is well-known in patients with vestibular deficits, in whom it indicates an overreliance on visual cues in sensory integration. Considering that optokinetic after-nystagmus (OKAN) reflects the response of the central network integrating visual and vestibular self-motion signals (velocity storage network), we investigated OKAN in 71 patients [17 (23.9%) females, 30.36 ± 9.05 years old] who suffered from persistent symptoms after a concussion and presented clinical signs suggesting visual dependence. Data were retrospectively compared with 21 healthy individuals [13 (61.9%) females, 26.29 ± 10.00 years old]. The median values of the slow cumulative eye position and of the time constant of OKAN were significantly higher in patients than in healthy individuals (slow cumulative eye position: 124.15 ± 55.61° in patients and 77.87 ± 45.63° in healthy individuals—*p* = 0.012; time constant: 25.17 ± 10.27 s in patients and 13.95 ± 4.92 s in healthy individuals—*p* = 0.003). The receiving operating curve (ROC) estimated on the time constant had an overall area under the curve of 0.73. Analysis of the ROC suggests that a test measuring the OKAN time constant could obtain a sensitivity of 0.73 and specificity of 0.72 in determining the origin of the visual-related disturbances in those patients (threshold 16.6 s). In a subset of 43 patients who also performed the Sensory Organization Test (SOT), the proposed OKAN test was twice as sensitive as the SOT. This study suggests that concussed patients with persisting visual symptoms may have an underlying impairment of the velocity storage mechanism and that measuring the OKAN time constant can objectify such impairment.

## Introduction

Concussion is currently a clinical diagnosis based on symptom assessment and clinical findings [1, 2]. In the context of traumatic brain injury (TBI), it is allocated to mild TBI. Since a concussion is classified as a transient functional rather than structural injury it is important to objectify clinical findings for the appropriate therapeutic approach [1, 2]. Growing evidence supports the assessment of eye movement abnormalities in the clinical evaluation of patients after the head impact [3–5]. The possibility to quantitatively document reported visual symptoms by eye movement assessment drives the linkage between ocular motor and concussion research [3, 6].

The vestibular/ocular motor screening (VOMS), proposed in 2014 by Mucha et al. [7], was the first structured assessment to focus on the vestibular and ocular motor systems after the concussion. VOMS, however, is based on the subjective evaluation of symptoms provoked by a sequence of different tasks. Recently, various quantitative eye movement tests have been evaluated. Of those, preliminary data that show promising discriminatory power in concussed patients, suggest a potential high diagnostic value following head impact [6]. For example, a simple saccade test could discriminate traumatic brain injury (TBI) patients from healthy control subjects with a sensitivity of 0.64 and a specificity of 0.65 in a cohort of 195 participants (144 patients, 51 control subjects) [8]. A composite test, sequentially evaluating the ocular motor response, vestibular function, and reaction time was reported to achieve a sensitivity of 0.76 and a specificity of 0.96 for SRC in a cohort of 220 participants (50 patients, 170 healthy control subjects) [9]. In this study, the major differences between concussed patients and healthy control subjects were found among the ocular motor parameters. Considering that approximately half of the brain circuits are involved in vision or ocular motor control, the sensitivity of vision-/eye movement-based tests to conditions after head impact is not surprising [3]. Such accurate assessment following a head impact, however, is challenging since different domains (e.g. neurocognitive, vestibular, neuroauditive, ocular motor, pain, emotional, autonomous) may be affected and currently, no clinical test or biomarker can make the diagnosis in isolation [10]. Accordingly, assessments of eye movement abnormalities should be always combined with accurate clinical evaluation and not considered a stand-alone screening tool. Similarly, comparing the efficacy of the aforementioned tests in patients with various clinical evaluations may help to correctly frame the use of these tests.

Eye movement assessments play an important role in the differential diagnosis of dizziness [11]. Occurrence, or worsening, of dizziness during exposure to environments with complex visual stimuli (e.g. supermarket aisles, busy crossroads) is commonly reported by concussed patients [12, 13]. Such symptoms are well known in patients with vertigo of various aetiologies and have been defined as a syndrome of visual vertigo [14] or visually induced dizziness (VID) [15]. Visual vertigo and VID imply a dependence of the patient on visual cues for orientation and self-motion [16]. To date, the evaluation of such visual dependence is mainly based on questionnaires [17, 18]. Current attempts to objectify visual dependence rely on indirect assessment, based on measuring postural sway or assessing the performance in perceptual tasks such as the subjective visual vertical [16, 19, 20].

It has been hypothesized that visual dependence is related to an alteration of the weighting of the sensory cues in the central processing network that generates a sense of orientation [14, 16]. Motor readouts of the activity of this network during the processing of full-field visual motion stimuli are optokinetic nystagmus (OKN) and after-nystagmus (OKAN) [21, 22]. OKN is a reflexive eye movement driven by the motion of the visual field on the retina, so-called retinal slip [23]. It consists of nystagmus with slow phases in the same direction as the moving stimulus and quick phases in the opposite direction. During natural full-field visual stimulation, the nystagmus is a combination of OKN and smooth-pursuit eye movements. The smooth pursuit eye movement system allows to voluntarily track moving targets by keeping their image on the fovea. If during an optokinetic stimulus, the light of the room is suddenly extinguished, the nystagmus continues for a while. As the smooth pursuit component stops within seconds, the velocity of the slow phases rapidly drops to then slowly decays to zero. The latter phenomenon is known as OKAN and can only be properly measured under laboratory conditions using large visual stimuli (e.g. optokinetic drums [23–25]) to drive the OKN before extinguishing the light. It is currently believed that the command signal for the OKN is generated through the central processing network integrating visual, vestibular, and proprioceptive information to provide an estimate of self-motion. This network, traditionally known as the velocity storage mechanism (VSM), can be characterized by a single time constant, i.e. a parameter measuring the time it takes to discharge a loaded velocity input. OKAN is a direct expression of the velocity storage and it can be characterized by the VSM time constant and gain, weighting the VSM contribution in the overall OKN response. OKAN, however, is rarely tested, as it is considered poorly localizing [24] and its measurements show high trial-to-trial variability [25].

Considering the reports on concussed patients who present symptoms compatible with visual dependence [12, 13], we hypothesized that in selected concussed patients with persisting, strong dizziness after visual stimuli the VSM is overacting when it extracts self-motion information from visual cues. To evaluate this hypothesis, we analysed OKAN of patients who showed signs and symptoms supporting impaired central integration of visual information after a concussion.

## Materials and methods

### Subjects

Data from 71 concussed patients (17 females, 30.4 ± 9.1 years old, mean ± standard deviation) whose evaluation at the Swiss Concussion Center (SCC) included OKAN testing between 01/01/2016 and 31/12/2018 were analyzed. Neurologists at the SCC ordered OKAN testing whenever a concussed patient suffered persistent visual-vestibular symptoms suggesting an increased visual dependence. The time span between head impact and OKAN testing varied between 6 and 4172 days (363 ± 629 SD). Standard CT or MR imaging in these patients were negative for traumatic lesions. In addition to the neurological examination, the patients were also assessed with the Sport Concussion Assessment Tool, 5th edition (SCAT5). In 69 patients a battery of laboratory vestibular tests, including video-head impulse test (vHIT), cervical and ocular vestibular evoked myogenic potentials (cVEMP, oVEMP), fundus photography of ocular cyclotorsion, and subjective visual vertical (SVV) was performed.

Depending on their clinical presentation, patients also performed the Sensory Organization Test (SOT) using the Neurocom Equitest® (NeuroCom International, Inc, Clackamas, OR, USA). Postural data were analysed by extracting the scores used by the Neurocom® system for clinical application [26, 27]

Data from 21 healthy individuals (13 females, 26.29 ± 10.00 years old) without a history of concussion were acquired with the same OKAN-paradigm and were used as control values.

Written informed consent for retrospective use of data was obtained from all participants. The protocol was approved by the local ethics committee Protocol Nr 2017–01208 and was in accordance with the ethical standards laid down in the Declaration of Helsinki for research involving human subjects.

### Experimental setting

**Fig. 1:**
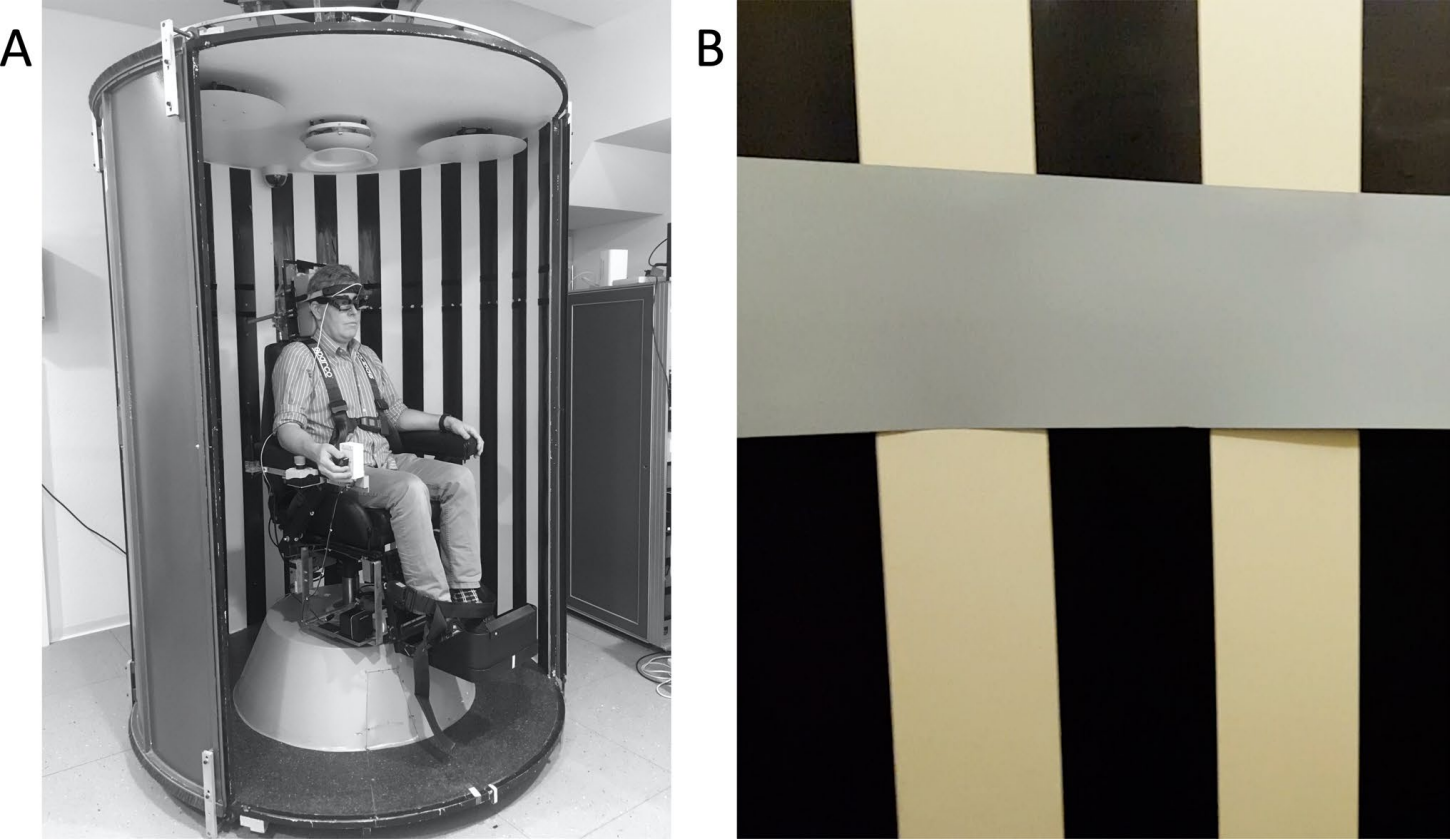
The experimental set-up. **a** The optokinetic drum before mounting the horizontal gray band and before closing the two sliding doors that will complete the cylinder. **b** A detail of the horizontal gray band mounted at eye level (from inside)

All recordings were obtained on a chair mounted on a two servo-controlled motor-driven axes turntable system (Tönnies D561, Freiburg, Germany; control system: Acutrol® ACT2000, Acutronic, Switzerland Ltd.). One axis rotates the chair and the other a cylinder (optokinetic drum, radius: 74 cm) mounted concentrically to the chair (Fig. 1). Remotely controlled LEDs are attached to the cylinder at the level of the subject’s eyes and ± 10° vertically. Safety belts around the feet and the shoulders prevent the subject from moving. An adjustable chin rest, a forehead strap, and pillows are used to stabilize the subject’s head. The inner wall of the drum was covered with 10 cm alternating black and white stripes, each subtending 7.7° of visual angle. The pattern filled the entire visual field. A horizontal grey band at eye level, extending 8° vertically, overlapped the stripes at the participants’ eye level and served to prevent foveal fixation and minimize the contribution of smooth ocular pursuit. Horizontal eye movements were recorded at 220 Hz with a head-mounted video-oculography (VOG) device (EyeSeeCam from EyeSeeTec, Munich, Germany) consisting of goggles with two mounted infrared cameras [28].

### Experimental procedure

The first trial was used to calibrate eye movements. Participants were asked to visually fix on a red LED and to follow it with the eyes without moving the head. The LED was initially positioned straight ahead at eye level and was moved to horizontal eccentricities from 25° left to 25° right and to vertical eccentricities from 10° up to 10° down.

Subsequently, the participants were exposed to two test trials whereby an optokinetic stimulus was delivered by rotating the drum for 30 s at 50°/s. At the beginning of each trial, the participants were in total darkness while the drum accelerated. Once the speed plateau was reached, the light was quickly turned on, initiating the optokinetic stimulus. Participants were instructed to keep the gaze within the horizontal grey band in front of them for the duration of the stimulus. The examiner controlled the eye video and position trace in real-time to ensure the participants' compliance. This procedure was aimed at facilitating stare-type optokinetic nystagmus (i.e. the participant is instructed to simply stare, as opposed to the look-type optokinetic nystagmus where the participant actively tracks the single stripes) maximising the contribution of the optokinetic system over the smooth pursuit system. After 30 s, the light was terminated and the OKAN was recorded for a minimum of 30 s or until the nystagmus stopped. The two trials were separated by a pause lasting a minimum of 60 s with the lights on, and the drum not moving, thus, allowing the discharge of any potential residual velocity storage activity.

### Data analysis

Data analysis was performed using MATLAB (The MathWorks, Natick, MA), release R2019b. Quantification of symptoms among the tested patients was performed by extracting two scores from the SCAT5 symptom questionnaires: the number of symptoms (min 0, max 22), i.e. the sum of symptoms with severity greater than 0, and the symptoms severity (min 0, max 132), i.e. the sum of the severity scores provided for each symptom. The vestibular function was quantified by means of 18 outcome variables obtained from the standard laboratory assisted vestibular tests and chosen according to clinical guidelines (see Table 2 for details).

Raw eye position data recorded by the EyeSeeCam® software during the OKAN tests were further processed. Velocity traces were obtained as the derivative of horizontal eye position traces using a 1st derivative Savitzky-Golay smoothing filter (with 2nd order polynomials and 9-point window size). Saccades and blinks were removed and missing data points were interpolated. The time when the light was turned off, i.e. the instant when the optokinetic stimulus terminated and the optokinetic after nystagmus (OKAN) started, was automatically identified from the turntable-control signal synchronized with the eye movement recording. This instant was defined as the time zero and only the eye velocity after this time point was used in the subsequent analysis. Two independent analysis were performed on the resulting velocity traces.

### Slow cumulative eye position analysis

The slow cumulative eye position (SCEP) was calculated as proposed by Hain and Patel [29]. The integral of the slow phase velocity was computed according to the trapezoidal rule, excluding the first 2 s to reduce impact of the smooth pursuit component on the integral. The missing seconds were accounted for adding the estimate of initial OKAN velocity multiplied by two. The mean velocity across the second after the lights were extinguished was used as an estimate of the initial OKAN velocity. The SCEP of each subject was calculated as the average of the slow cumulative eye position calculated from the two trials.

### Model-based analysis

Eye velocity traces were fitted using a two-exponential model (Eq. 1) [29–31]: a rapid one, taking into account the initial drop of the smooth-pursuit component evoked by the stimulus; and a slower one, accounting for the OKAN. The analysis focused on the second exponential.

1 $$ V\left( t \right) = \underbrace {{g_{sp} e^{{{\raise0.7ex\hbox{${ - t}$} \!\mathord{\left/ {\vphantom {{ - t} {T_{sp} }}}\right.\kern-\nulldelimiterspace} \!\lower0.7ex\hbox{${T_{sp} }$}}}} }}_{smooth - pursit} + \underbrace {{g_{OKAN} e^{{{\raise0.7ex\hbox{${ - t}$} \!\mathord{\left/ {\vphantom {{ - t} {T_{C} }}}\right.\kern-\nulldelimiterspace} \!\lower0.7ex\hbox{${T_{C} }$}}}} }}_{OKAN}, $$

In Eq. 1, $V(t)$ is the fit velocity divided by the speed of the optokinetic drum (i.e. 50°/s), ${g}_{sp}$ and ${T}_{sp}$ are, respectively, the gain and the time constant of the smooth-pursuit component and ${g}_{OKAN}$ and ${T}_{C}$ those of the OKAN components of the response. During the non-linear least square fit procedure, the values of gains were bounded between 0 and 1, those of ${T}_{sp}$ between 0 and 2 s [29–31] and those of ${T}_{C}$ between 2 and 100 s. If the r^2^ of the fit was lower than 0.5, the resulting parameters were excluded from the subsequent analysis. This resulted in discarding one of the two trials from 14 patients (19.7%) and 5 healthy participants (23.8%) and both trials (i.e. the entire data of the participant) from 10 patients (14.1%) and 3 healthy participants (14.3%). When the parameters fitted from both trials of a participant were retained, they were averaged, so that each subject was associated with a single gain and a single time constant. The resulting time constants were considered as the outcome of the OKAN test and compared with the healthy control group in the statistical analysis.

In addition, the results of the patients (*n* = 43) that performed the Equitest® before or up to ten days after the OKAN testing were separately analysed in a one-to-one comparison of the SOT and the OKAN test outcomes. This time frame was selected to minimize the risk that a higher sensitivity of the OKAN could be due to intervening significant improvements in the patient condition. According to standard clinical procedures [27], the equilibrium scores of the SOT were used to generate a four-sensory analysis ratio (SAR) describing how well subjects are able to use somatosensory (SOM), visual (VIS), and vestibular (VEST) systems for balance control and how they rely on visual cue even if visual information is not reliable (PREF) (see Table 1 for details).

**Table 1:** Sensory organization test conditions

Condition	Visual feedback	Somatosensory feedback
SOT 1	Eyes open	Fixed platform
SOT 2	Eyes closed	Fixed platform
SOT 3	Sway-referenced visual surround	Fixed platform
SOT 4	Eyes open	Sway-referenced platform
SOT 5	Eyes closed	Sway-referenced platform

SAR		Description
SOM	$\frac{SOT2}{SOT1}$	Ability to use somatosensory cues
VIS	$\frac{SOT4}{SOT1}$	Ability to use visual cues
VEST	$\frac{SOT5}{SOT1}$	Ability to use vestibular cues
PREF	$\frac{SOT2+SOT5}{SOT3+SOT6}$	Visual preference, i.e. propensity to use visual feedback even when inaccurate

### Statistical analysis

To test whether the SCEP and the model parameters were normally distributed, a Lilliefors test was performed. Since normality was not verified, a non-parametrical test (Wilcoxon rank-sum test) was used to compare the median of SCEP, gain, and time constant distributions estimated from OKAN patients and healthy participants.

Receiving operating characteristic (ROC), area under the curve, and sensitivity and specificity were calculated to estimate the capacity of a test measuring OKAN time constant to objectify visual dependence or visually-induced vertigo in patients who suffered head impact versus healthy participants. In this process, we determined optimal factor settings with the idea of privileging specificity on sensitivity (relative weights in determining the cut-off value 0.7 and 0.3, respectively; this assumption was made to counterbalance the different sample size between the patient and control group). Kendall’s Tau (*τ*_k_—a non-parametrical correlation index, which does not require the hypothesis of linearity) was used to evaluate the possible relations between the estimated time constant and the following variables: time passed between the head impact and the test date, age at test date, SCAT5 severity score and number of symptoms.

## Results

The mean SCAT5 severity score of the 71 patients was 37.16 ± 26.04 SD and the mean number of symptoms was 13.02 ± 6.34 SD, confirming the severity of symptoms in the range expected for a concussed population [32, 33]. Automatic analysis of the outcomes of laboratory vestibular tests found that 58% of the patients had a result outside the normative range in at least one outcome variable (40 out of 69 patients who underwent laboratory vestibular tests) but only 23% (16 out of 69) had pathological value in one outcome variable from more than one test (see Table 2). To control for false positive in the automatic analysis, the entire test battery of each patient was evaluated by a neurologist who indicated that ~ 54% (37 out of 69) can be considered with a pathological outcome of the vestibular test battery and that ~ 7% (5 out of 69) showed horizontal spontaneous nystagmus. The results are shown in Table 2.

**Table 2:**
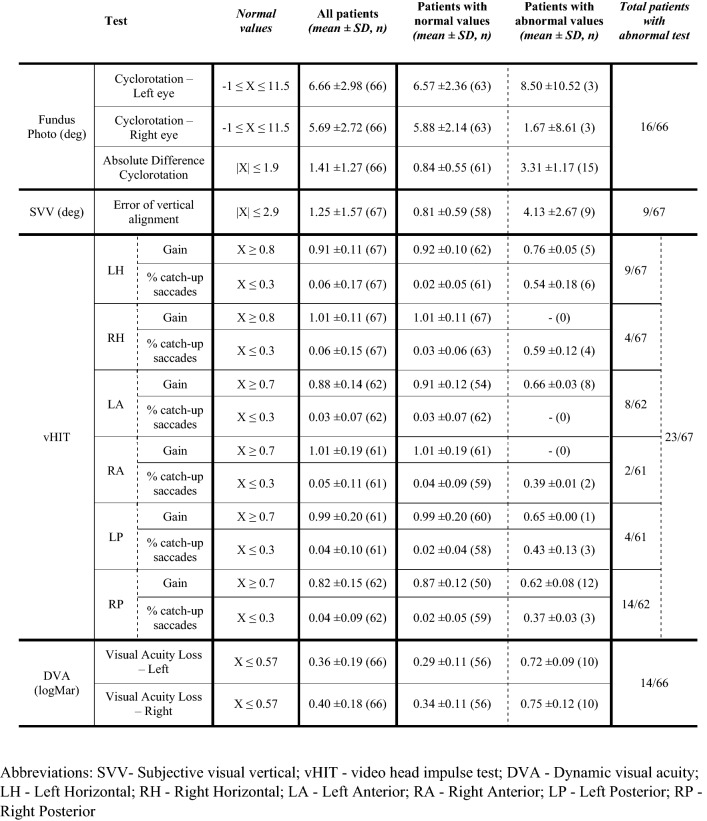
Data distributions of vestibular battery

*SVV* subjective visual vertical, *vHIT* video head impulse test, *DVA *dynamic visual acuity, *LH *left horizontal, *RH *right horizontal, *LA *left anterior, *RA *right anterior, *LP *left posterior, *RP *right posterior

The mean SOT sensory analysis ratios (SAR) were 0.94 ± 0.06 SD for the somatosensory system (SOM), 0.83 ± 0.13 SD for the visual system (VIS), 0.57 ± 0.20 SD for the vestibular system (VEST) and 0.98 ± 0.12 SD for visual preference (PREF).

### Optokinetic after-nystagmus (OKAN)

**Fig. 2:**
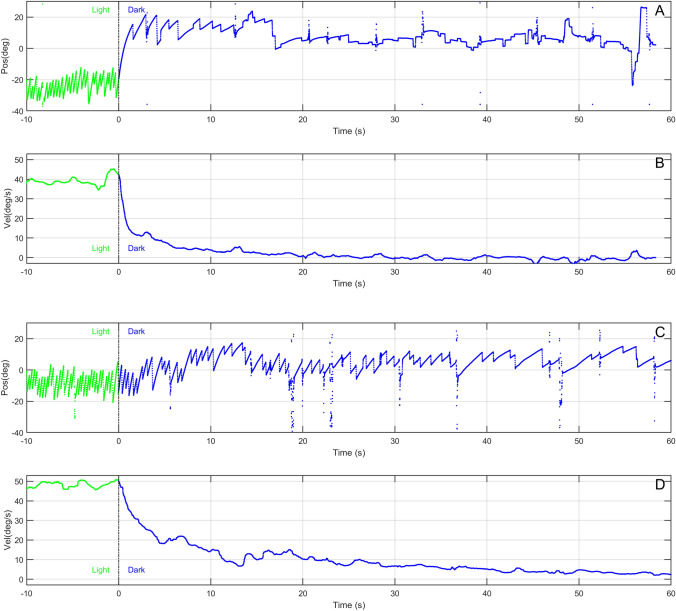
Comparison of the eye movements data in one healthy participant (**a**, **b**) and a patient (**c**, **d**). Eye position (**a**, **c**) and slow phase velocity (**b**, **d**) are depicted as a function of time. The vertical lines corresponding to the 0 in the time vectors represent the instants when the light was switched off and separates the response observed in light (green) and the response observed in darkness (blue)

**Fig. 3:**
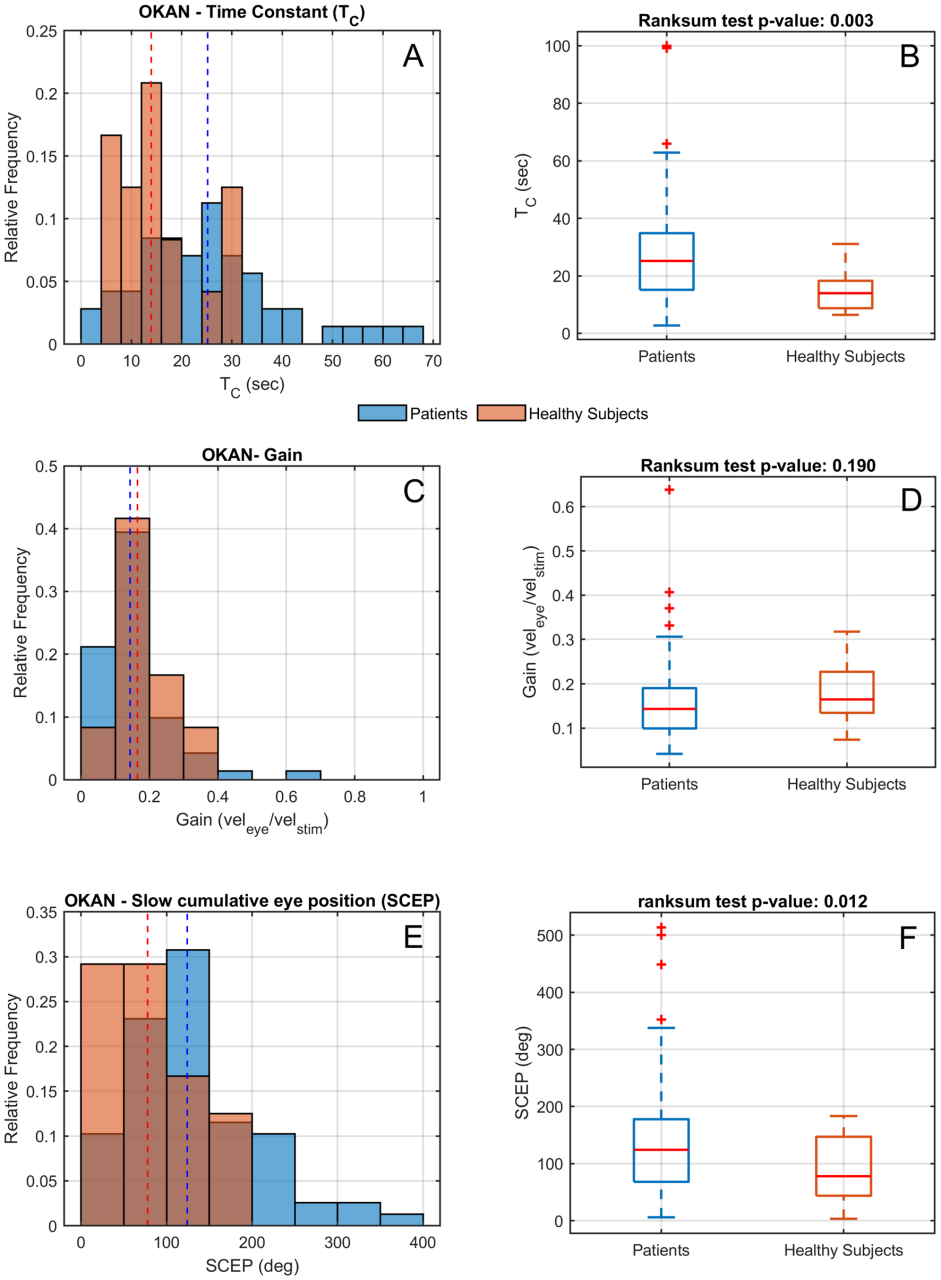
Histograms of relative frequency (**a**, **c**, **e**) and box plots (**b**, **d**, **f**) of time constants (**a**, **b**) and gains (**c**, **d**) and slow cumulative eye position—SCEP—(**e**, **f**) of OKAN in patients (blue) and healthy controls (orange). The vertical dashed lines in the histograms represent the median of the patients (blue) and of the healthy participants (red)

**Fig. 4:**
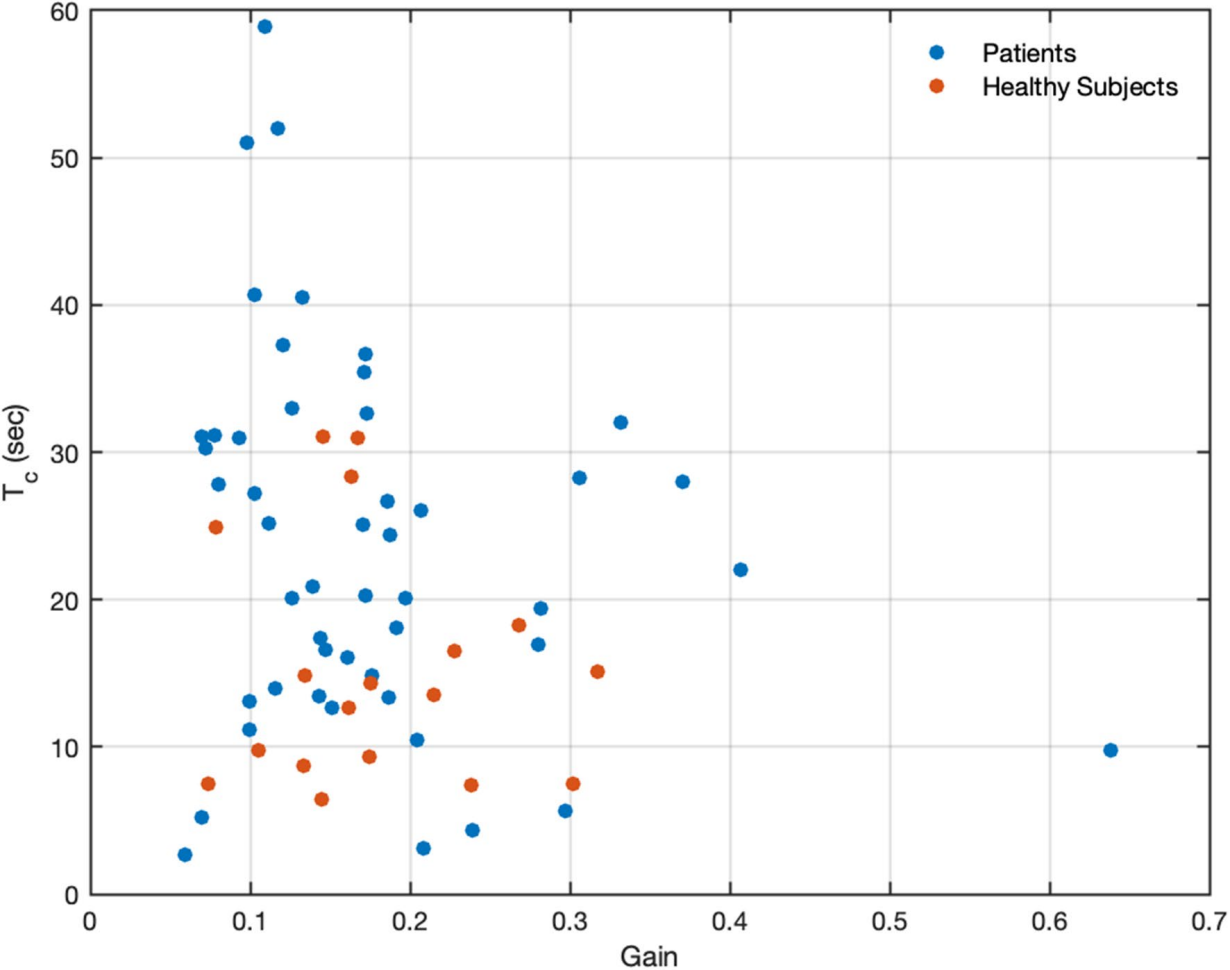
Scatter plot of showing the position of each participant (patients—blue dot; healthy controls- red dots) in the parameters' space (gain vs time constant)

Figure 2 depicts eye position and eye velocity as a function of time, recorded in a healthy participant (panel A and B) and in a patient showing an abnormal response (panel c and d) during the last 10 s of optokinetic stimulation (light) and the first 60 s after the light was turned off (dark). OKAN is clearly discernible in the second part of both position traces. The decline of the corresponding slow-phase velocity traces can effectively be described by an exponential decay (Fig. 1). The velocity traces demonstrate that, after the initial rapid drop due to the cessation of the smooth pursuit component, OKAN slow phase velocity decays in the two subjects with different dynamics. The decay occurs much more rapidly in the healthy participant and the velocity approaches zero after roughly 30 s, corresponding to a time constant in the order of 7.45 s (a value in accordance with the literature [25]). The eye velocity of the patient, instead, shows a much slower decline with a time constant of 28.01 s and therefore a residual drift is still present after 60 s. This observation was confirmed in the whole population by both analyses (Fig. 3). Pooling data, median SCEP was 124.15 ± 55.61 (MAD—median absolute deviation) degree in the patients' group, significantly larger (Wilcoxon rank-sum test: *p* = 0.012) than the one calculated from the healthy subjects' data (77.87 ± 45.63 MAD degree). The median time constant of patients was 25.17 ± 10.27 (MAD) seconds, while the median time constant of healthy subjects was 13.95 ± 4.92 (MAD) seconds. Thus, the time constants of the patients were significantly longer than those of healthy subjects (Wilcoxon rank-sum test: *p* = 0.003). No difference was instead observed for the gains, i.e. the ratio of the initial OKAN amplitude to the OKN stimulus velocity (*p* value = 0.190; patients median gain: 0.14 ± 0.04 MAD; healthy participant median gain: 0.16 ± 0.04 MAD). The individual positions of patients and healthy controls in the parameter space (gain-time constant plot) are shown in Fig. 4.

**Fig. 5:**
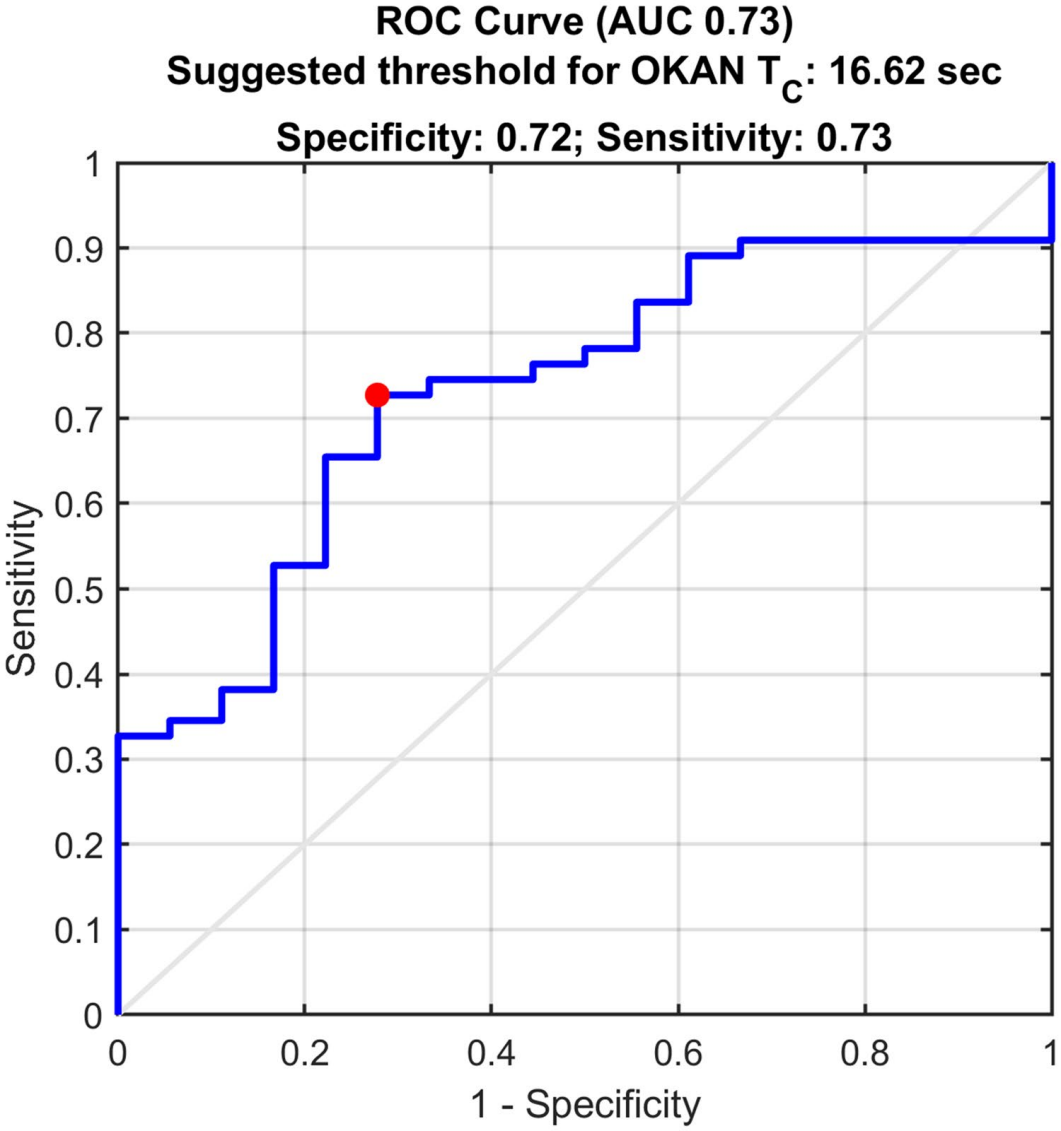
Receiving operating curve calculated on our dataset for the capacity of a test based on OKAN time constant to distinguish healthy subjects from patients who suffered head impact and present with signs and symptoms of visual dependence

The ROC curve computed using all the time constants estimated from the patient and control groups had an overall area under the curve of 0.73 (Fig. 5). Weighting the sensitivity less than the specificity (0.3 and 0.7, respectively), we estimated an optimal operating point of the ROC curve equals to 16.6 s, obtaining a sensitivity of 0.73 and specificity of 0.72. This implied that using 16.6 s as the threshold value, a test measuring the OKAN time constant has an accuracy of 0.73 in separating healthy subjects from patients who suffered head impact and present with signs and symptoms of visual dependence.

The OKAN time constants measured in the patients did not correlate with the time passed since the head impact or with age (*τ*_k_ = 0.01, *p* value = 0.99 and *τ*_k_ = 0.17, *p* value = 0.07, respectively). No significant differences between the two sub-populations of patients with OKAN time constant above and below the 16.6 s threshold were observed in the time passed since the head impact [*T*_C_ < 16.6 s (100 ± 71, median ± MAD) vs *T*_C_ ≥ 16.6 s (124 ± 105); *p* value = 0.481] or in age [*T*_C_ < 16.6 s (30.32 ± 4.56, median ± MAD) vs *T*_C_ ≥ 16.6 s (30.66 ± 3.66); *p* value = 0.293]. Similarly, the OKAN time constants did not correlate with the SCAT5 severity score or the number of symptoms (*τ*_k_ = − 0.10, *p* value = 0.35 and *τ*_k_ = − 0.11, *p* value = 0.35, respectively). No significant difference was observed for these variables between the subgroups of patients with time constant above-threshold and below threshold [severity score: *T*_C_ < 16.6 s (44 ± 16, median ± MAD) vs *T*_C_ ≥ 16.6 s (39 ± 19); *p* value = 0.601; number of symptoms: *T*_C_ < 16.6 s (15.5 ± 2.5, median ± MAD) vs *T*_C_ ≥ 16.6 s(15.0 ± 4.0); *p* value = 0.481].

### Comparison between the assessment of OKAN time constant and the assessment of the use of visual cue in a balance task (SOT)

**Fig. 6:**
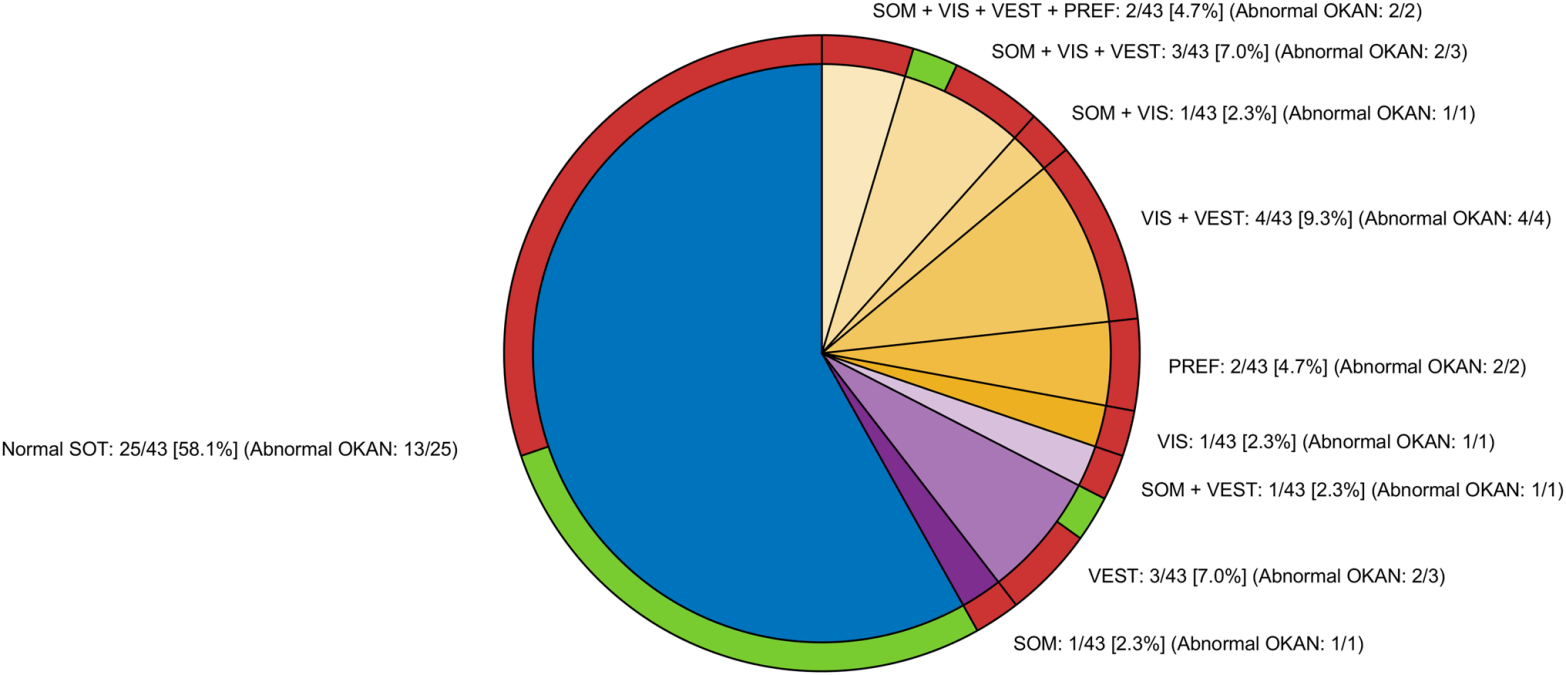
Combined pie chart comparing the results of the SOT (inner circle) to the one of the OKAN test (outer circle) in our patients. For the SOT, the blue sector represents the patients with normal results, the yellow sector those with a pathological result in VIS, PREF or both (see Table 1 for SOT scores definitions), and the pink sector those with a pathological SOT result not including VIS or PREF. For the OKAN, the green sectors represent patients with a normal result and the red sectors those with an abnormally long time constant. The OKAN sectors (outer circle) are also separated to match the SOT sectors and patients in each outer sector correspond to patients in the respective inner sector. This allows a direct comparison between patient classification from SOT and OKAN test

In 43 patients (i.e. the subset of patients that performed the Equitest® before or up to ten days after OKAN testing) the results of the SOT and the OKAN testing were compared. When interpreting the SOT results, all patients with insufficient results in PREF or in VIS (i.e. patient with any combination of results containing insufficiency in these, subsequently named VIS/PREF group) were considered as positive (i.e. as cases where SOT results identified a dysfunction in relation to visual cues). As shown in Fig. 6, the VIS/PREF group consisted of only 30% of patients (*n* = 13; yellow shaded pie sectors), while 58% (*n* = 25) had a normal SOT result (blue pie sector) and ~ 12% (*n* = 5) were categorized with SOM, VEST or both impairments (violet shaded sectors). In contrast, 68% of patients (*n* = 29) had abnormally long OKAN time constant above 16.6 s, i.e. the threshold defined with the ROC analysis (see red pie sectors in Fig. 6).

**Fig. 7:**
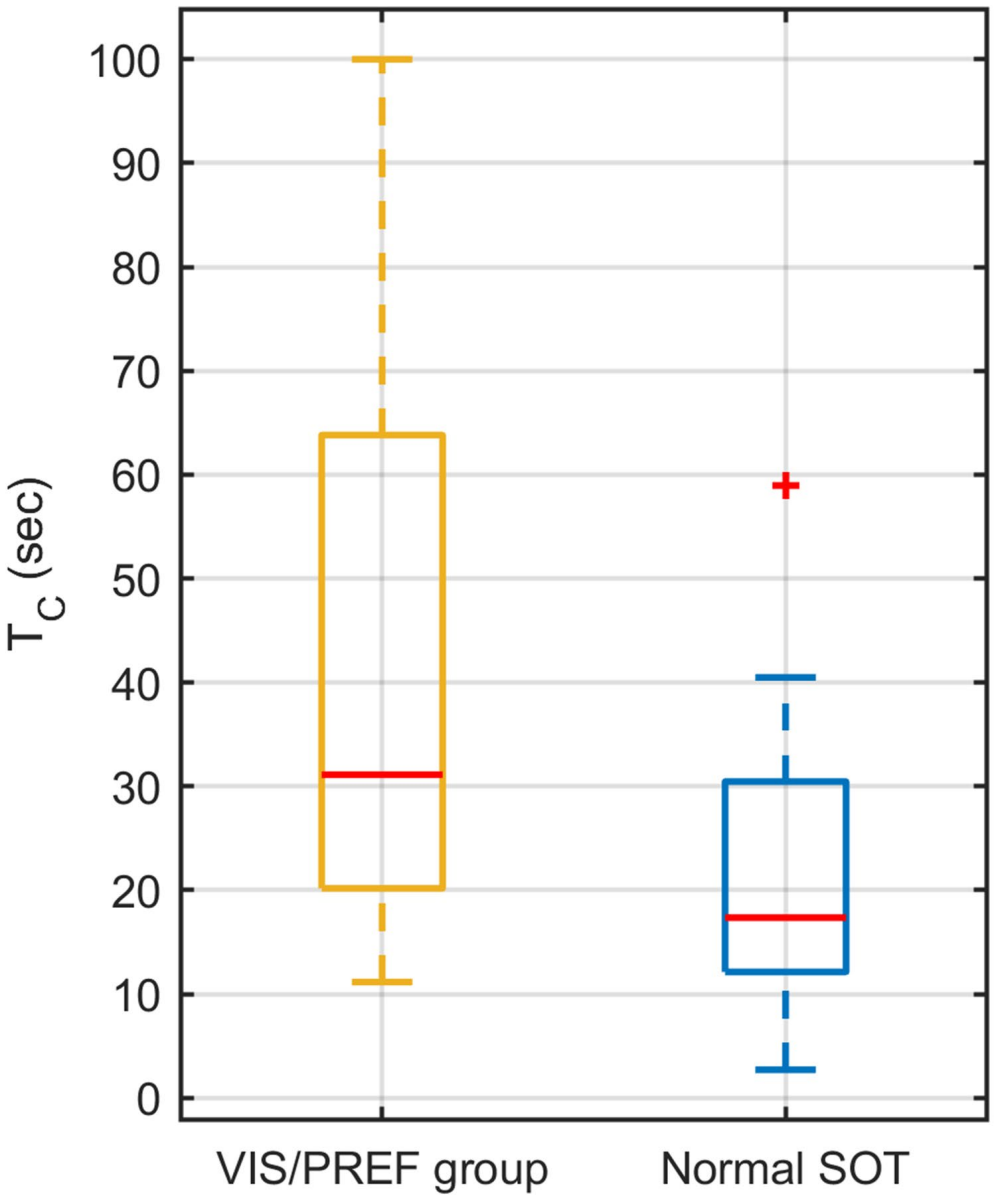
Box plot of the OKAN time constants in the patients in the VIS/PREF group according to the SOT (yellow) and in the patients classified normal by the SOT (blue)

It is noteworthy that all but one patient (12/13) in the VIS/PREF group showed an abnormally long OKAN time constant. The median OKAN time constant of the VIS/PREF group was 31.13 ± 14.50 MAD seconds (median ± MAD), which was significantly higher (Wilcoxon rank-sum test: *p* = 0.013) than the median time constant of 17.36 ± 8.70 MAD of the patient considered normal by the SOT (Fig. 7).

## Discussion

The present study demonstrated that an abnormally prolonged OKAN time constant can identify patients who show signs and symptoms of impaired central integration of visual information following a head impact.

An abnormally high OKAN time constant, as found in our patients, implies an alteration in the dynamical properties of the central integrator of self-motion cues (i.e. the velocity storage mechanism). Thus, a majority of concussed patients with visual vertigo may suffer from the consequences of an impaired velocity storage mechanism. The brain supposedly estimates head velocity as the sum of the direct signal from the semicircular canals and its integral from the velocity storage [21]. This operation improves the semicircular canals estimate of head velocity. As the integrator is leaky, the range of frequencies affected by the improvement has a lower bound that depends on the value of the time constant. It has been proposed that the leakiness of the integrator is not a limitation, but a functional property [34, 35]. A perfect integration, which would result in a perfect estimate of head velocity, will inevitably accumulate biological noise. This functional leak is compensated by feeding the integrator with the self-motion cues extracted from the visual system (i.e. congruent slip of visual field on the retina), so that each sensory information is weighted in an optimally tuned balance [35].

An abnormally long time constant of the integrator suggests that the functional equilibrium between vestibular, visual (and others) cues is shifted. This shift can have different effects depending also on the other parameters controlling how visuo-vestibular stimuli are fed to the velocity storage. An abnormally long OKAN time constant could also provide a direct explanation of why the tested patients complain of symptoms and unsteadiness induced by complex visual stimuli, so-called visual dependence. Busy crossroads, walking on the patterned floor or through supermarket aisles provide low-frequency visual motion signals that are integrated by the velocity storage. A prolonged time constant of the OKAN implies that this information requires an abnormally long time to discharge, thus perpetuating self-motion illusion induced by these visual stimuli. This interpretation is supported by a similar finding of an “above average” OKAN time constant in healthy individuals prone to visually induced motion sickness [36].

Analysis of the receiving operating curve (ROC) estimated on our data evidenced that a test measuring the OKAN time constant with a threshold of 16.6 s obtained a sensitivity of 0.73. Such test was also highly specific as roughly 1 in 5 healthy individuals (specificity = 0.72) had a time constant longer than 16.6 s. Although the number of healthy controls in this study was limited, the recorded values could be considered trustworthy as the distribution of the time constant recorded in our healthy group was not statistically different from the one of Tijssen et al. [25], the only study presenting a large OKAN data set from healthy humans. To account for the limited number of healthy controls in our study, we considered the OKAN time constants measured by Tijssen et al. [25]. Applying the threshold defined on our data (i.e. 16.6 s) on their dataset, the specificity amounts to 0.92. It is therefore safe to assume that the estimate of the current study is conservative.

OKAN is rarely tested in the clinical routine. This is due to the following concerns: (1) the unclear interpretation of OKAN alterations and limited localizing value [24]; (2) the elevated trail to trial variability of the OKAN response [25, 29].

The presented data, however, suggest a direct clinical interpretation: a prolonged OKAN identifies concussed patients where visually triggered symptoms originate from an impaired velocity storage mechanism [23]. This also implies a localizing value, as these patients will have impairments (or alterations) at the level of the cerebellum, where the activity of the velocity storage is supposedly controlled. Previous studies have shown prolonged horizontal OKAN after removal of the nodulus/uvula [37].

The trail to trial variability of OKAN is unlikely to significantly reduce the usefulness of testing OKAN in this population of patients. It has been demonstrated that a reliable estimate of the OKAN time constant requires averaging many repetitions [25], and analysis has been shown to be challenging [29]. On the contrary, the presented data originates from only two repetitions of a 1–2 min trial, and eye slow phase velocity of OKAN is fitted with a single exponential model (with a second exponential accounting for smooth-pursuit). The high specificity and sensitivity achieved, however, do not imply that the time constants we estimated were highly accurate, but that the alteration of the time constant in patients is sufficiently large that the test is sensitive and specific even with noisy data. It is possible that, by increasing the number of repetitions, specificity and sensitivity will further improve. On the other hand, several patients were substantially strained by the test, as it exacerbated the symptoms. Increasing the number of trials may, therefore, be unpractical or even detrimental for the patients' collaboration.

One problem that may be behind the limited use of OKAN testing is the lack of a proper set-up to generate a reliable response. It is established that testing OKAN properly requires a large portion of the visual field being stimulated [23, 30] by a coherent visual motion that reliably replicates a rotating environment [23, 38, 39]. This is achieved optimally with the patient in a physical optokinetic drum, but various experiments demonstrated that measurable OKAN can be obtained with different setups, ranging from virtual reality [36, 40] to a large curved screen [38, 39]. While we did not test these setups, it can be assumed that as long as they successfully elicit OKAN it will not affect the time constant. As a poor setup may generate weak OKAN, a decrease in the reliability of the estimated time constant may occur, and thus set-up specific normative values may be necessary to set an adjust threshold.

### Comparison between OKAN test and other clinical tests that measure impaired integration of visual cues

Visually triggered symptoms suggesting problems in central integration of visual cues are relatively common in vestibular patients [9]. Their assessment is however usually based on the patient's history, possibly collected through validated questionnaires, and few tests have been proposed to measure them. The best known are the Sensory Organization Test (SOT) [26] and Rod and Disk test [19, 20, 41].

The SOT was performed by 70% (*n* = 43) of our patients that successfully completed the OKAN test. The comparison suggests a striking superior performance of the OKAN test. Using the SOT only 13 the patients showed a pathological outcome related to the use of visual cues (counting any patient with insufficient performance in PREF or VIS scores), corresponding to test sensitivity of ~ 30%. The sensitivity of the OKAN test was more than double (~ 67%) with 29 patients showing an abnormal OKAN time constant. This group included all but one patient with insufficient results in the SOT (see red sectors over the blue pie sector in Fig. 6). Notably, the patient with insufficient results in the SOT but normal OKAN presented impairment in almost all SOT scores. Therefore, the SOT did not specifically single out problems in integration of visual cues in this patient. The comparison of the time constants between patients of the VIS/PREF group and those considered by normal by SOT further corroborate the higher sensitivity of the OKAN test: the separation between the time constant of the two groups is indeed well above the OKAN test threshold of 16.6 s (Fig. 6).

The superior performance of the OKAN test in identifying a problem in the central integration of visual cue is not surprising. The SOT assesses multi-sensory integration in the context of a balance task and should not be interpreted as an overall measure of visual dependence. A failure in the integration of visual cues may be undetected by SOT as it is irrelevant for a balance task. Furthermore, as balance task requires complex motor coordination and may be affected by postural strategies, other reasons may affect the SOT sensitivity to sensory integration impairments [i.e. the features of the specific balance task tested by SOT or the subject-specific strategies developed to compensate for the deficit (e.g. elevated stiffness)]. The OKAN test measures a central oculomotor reflex that directly originates from the central process integrating visual cues, and therefore offers a direct measure of impairment in the processing of visual cues.

A test reported to reliably assesses visual dependence in vestibular patients [19, 20, 41] is the Rod and Disk test, first conceived by Dichgans et al. [41]. This test was not routinely performed in our clinic at the time the OKAN test data were collected, and therefore no direct comparison of the two tests could be performed. The Rod and Disk test is a behavioural test where the subject, in complete darkness, has to align to the perceived vertical a luminous line over imposed on a luminous disk that may induce a visual bias. The Rod and Disk test can therefore prone to cognitive bias, and its results are affected by the intensity of the symptoms and the collaboration of the patient. The OKAN test, measuring a central reflexive response, is free from these biases.

Altogether, our promising data suggest that a test based on OKAN has the potential to identify alteration of velocity storage in concussed patients presenting persisting visual symptoms and might help in defining therapeutic strategies in individual patients.

### Limitations and open research questions

The study tested OKAN in patients who suffered a concussion and, at the time of the test, presented persisting symptoms compatible with excessive visual dependence. As the aim of the study was to investigate whether the patients' OKAN was abnormal, the control group consisted of healthy participants. This choice does not allow to establish whether the observed abnormal OKAN time constant is a feature of any patient with visual dependence or if it is specific for concussed patients. While it might be possible that any patient with visual dependence (currently diagnosed within the PPPD group) has an abnormal OKAN, answering this question would have required an additional control group, consisting of visual dependent patients without concussion. Given the variety of precipitants that can lead to visual dependence, such control group would have been inhomogeneous, with the risk of confounding the current finding (e.g. if this group would have resulted not different from neither the patients nor the control). On the other hand, selecting only visually dependent patients with a specific precipitant would have not answer a general question on the relation between OKAN and visual dependence (as the study would have just compared the visual dependence that follows the selected precipitant and concussion). Future studies, comparing several visual dependent patients grouped by precipitants, will be necessary to answer this question.

Notably, the control and test groups were not gender matched. While gender is reported for completeness, it is not considered a relevant factor for the OKAN time constant [25]. This was therefore not accounted for in the statistical analysis to avoid multiplying factors without necessity.
